# Calreticulin Type 26 Mutation in Myelofibrosis: A Rare Variant With Diagnostic Challenges

**DOI:** 10.1002/jcla.70201

**Published:** 2026-05-13

**Authors:** Teresa Maltese, Giuseppina Raffa, Fabio Stagno, Rossana Leanza, Paola Barone, Alessandro Allegra, Teresa Pollicino

**Affiliations:** ^1^ Division of Advanced Diagnostic Laboratories, Department of Clinical and Experimental Medicine University Hospital “G. Martino” Messina Messina Italy; ^2^ Hematology Unit, Department of Human Pathology in Adulthood and Childhood “Gaetano Barresi” University Hospital “G. Martino” Messina Messina Italy

**Keywords:** CALR, calreticulin, calreticulin type 26, calreticulin uncommon variant, myeloproliferative neoplasm

## Abstract

**Background:**

Myeloproliferative neoplasms (MPNs) are clonal hematologic disorders commonly driven by mutations in *JAK2*, *MPL*, or *CALR*. Because routine CALR assays are largely optimized for the canonical Type 1 and Type 2 exon 9 variants, rare noncanonical mutations may be missed, creating diagnostic challenges.

**Methods:**

We report the case of a 78‐year‐old woman diagnosed with an MPN in 2016 and treated with hydroxyurea. Initial molecular testing included *JAK2* V617F screening and PCR‐based fragment‐length analysis by capillary electrophoresis for *CALR* exon 9 indels, both of which yielded negative results. After clinical progression, in 2024, repeat hematologic evaluation, bone marrow biopsy, Sanger sequencing, cloning, and next‐generation sequencing (NGS) were performed.

**Results:**

Disease progression was associated with marked thrombocytosis, leukocytosis, microcytic anemia, and bone marrow findings consistent with primary myelofibrosis. Advanced molecular testing identified a heterozygous CALR c.1122delG frameshift mutation (Type 26), a rare exon 9 variant not detected by the initial assay. Cloning confirmed heterozygosity, and NGS demonstrated a variant allele frequency of 43%. An additional CBL splice‐site mutation (VAF 17%) was also detected.

**Conclusion:**

This case highlights the limitations of routine assays focused on common CALR mutations and supports comprehensive sequencing‐based approaches for detecting rare CALR variants. Expanded molecular testing may improve diagnostic accuracy and clinical classification in MPNs.

Myeloproliferative neoplasms (MPNs) are a group of clonal hematopoietic stem cell malignancies characterized by excessive production of myeloid lineage cells [1]. These include polycythemia vera (PV), essential thrombocythemia (ET), and primary myelofibrosis (PMF) [2]. The most common molecular driver in MPNs is the *JAK2 V617F* mutation, present in > 95% of PV cases and 50%–60% of ET and PMF cases [3]. Mutations in the *MPL* gene, encoding the thrombopoietin receptor (TpoR) protein, are found in 3%–7% of ET and PMF cases [3]. Patients with ET or PMF lacking both *JAK2* V617F and *MPL* mutations typically harbor somatic mutations in the calreticulin (*CALR*) gene [4].

*CALR* mutations occur almost exclusively in ET and PMF cases, with rare incidences in myelodysplastic syndromes, myelodysplastic/myeloproliferative syndromes, and chronic neutrophilic leukemia [4]. As oncogenic drivers, *CALR* mutations are included among major diagnostic criteria for ET and PMF in the World Health Organization's hematological malignancy classification. Typically, patients with suspected ET or PMF undergo screening for *CALR* mutations after a negative result for *JAK2* mutations [5, 6].

The *CALR* gene, located on chromosome 19, consists of nine exons and encodes a highly conserved luminal Ca^2+^‐binding chaperone that plays a critical role in glycoprotein folding and intracellular calcium signaling [2, 4, 7]. In 2013, 36 *CALR* mutations were classified [4], and since then, 169 have been identified [8], all involving insertions or deletions. The two most common mutations, Type 1*/CALR*del52 and Type 2*/CALR*ins5 [2, 4, 7], account for 88.9% of cases [9] and are the ones detected by most commercially available diagnostic tests. These mutations produce novel C‐terminal amino acid sequences, disrupting the *KDEL* endoplasmic reticulum (ER)‐retrieval sequence (which acts as a sorting signal to ensure these proteins remain in the ER) and leading to altered calcium‐binding properties and pathogenic downstream effects [2, 8]. Mutant *CALR* proteins exhibit mislocalization to the cell surface, where they bind to the TpoR and activate *JAK/STAT* signaling [9]. Structural alterations due to these mutations result in a net increase in positively charged amino acids compared to the wild‐type (WT) protein (Figure 1) [2, 10]. Additionally, disrupted intracellular calcium mobilization from the ER triggers store‐operated calcium entry (*SOCE*), which is implicated in abnormal platelet production in mutant megakaryocytes [7].

**FIGURE 1:**
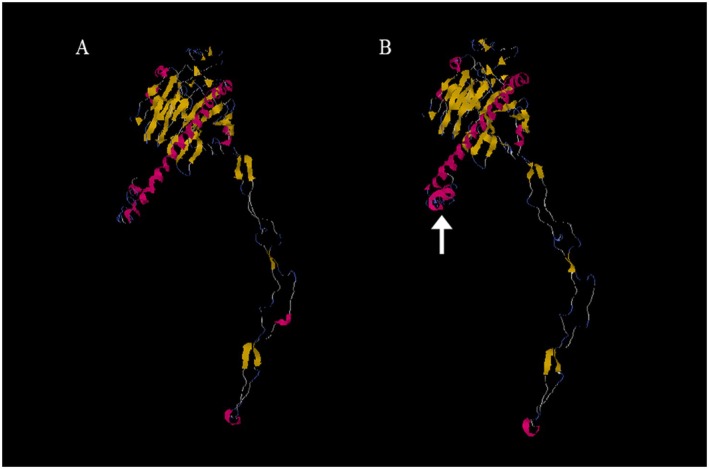
3D model protein structure prediction of the *CALR* protein, developed by I‐TASSER web server. (A) *CALR* wild type; (B) *CALR* Type 26 mutation.

We report the case of a 78‐year‐old woman diagnosed with a MPN in 2016, who was treated with Hydroxyurea (500 mg, two tablets daily). At presentation, an abdominal ultrasound revealed splenomegaly (14 cm), renal microlithiasis, and renal cysts. Laboratory tests showed a moderate elevation of brain natriuretic peptide levels (251 pg/mL) and increased inflammatory markers (C‐reactive protein > 20.9 mg/L). Investigations were also initiated to evaluate the patient's eligibility for potential Ruxolitinib treatment.

In 2016, she also underwent molecular testing for *JAK2* V617F and *CALR* exon 9 indels. *CALR* testing was performed using PCR‐based fragment‐length analysis with capillary electrophoresis, utilizing primers that flank exon 9 and target the two main indel hotspots. Sequence analysis was done using the GeneScan Analysis Software (Applied Biosystems). This test reliably detects indels with a variant allele frequency (VAF) of 5% or higher and is optimized for the common “Type 1, *CALR*del52” and “Type 2, *CALR*ins5” mutations. Smaller or low‐burden events, such as a 1 bp deletion below 5% VAF, may fall below the test detection threshold and be considered normal size variation. Both *JAK2* V617F and the two major *CALR* indels were reported as negative.

In 2024, clinical progression prompted repeat hematologic and molecular evaluation. Routine blood tests demonstrated marked thrombocytosis (1,564,000/mm^3^), leukocytosis (11,370/mm^3^), and persistent microcytic, hypochromic anemia. Bone marrow biopsy showed subcortical fibrotic remodeling, age‐adjusted hypercellularity due to granulocytic hyperplasia, and numerous, variably‐sized megakaryocytes—several exhibiting hypolobated, “cloud‐like” nuclei in loose clusters—as well as abundant hemosiderin‐laden macrophages and a well‐preserved erythroid lineage, consistent with PMF.

Repeat *CALR* testing by Sanger sequencing of exon 9 revealed a heterozygous single‐nucleotide deletion (c.1122delG), which resulted in a frameshift and a novel C‐terminal *CALR* sequence (see Figure 2A). This low‐frequency 1 bp deletion likely escaped detection in 2016 due to its presence below the 5% VAF limit of the fragment‐analysis assay and the fact that the assay design focused on larger, canonical indels. The mutation c.1122delG is an uncommon variant previously reported in the literature and designated as Type 26. Cloning and sequencing of PCR products confirmed the presence of both mutated (Figure 2B) and WT clones (Figure 2C), thereby establishing the heterozygous nature of the c.1122delG mutation. Next‐generation sequencing (NGS) further supported these findings, showing a VAF of 43%. NGS also identified a Casitas B‐lineage Lymphoma (*CBL*) mutation (c.1096IG>C splice site) with a VAF of 17%. Although categorized as a nondriver mutation in MPNs, the *CBL* mutation contributes to disease complexity by affecting disease progression and phenotypic heterogeneity [10]. Indeed, nondriver mutations, while not directly initiating the disease, contribute to its progression and variability by influencing the overall genetic and phenotypic landscape [6].

**FIGURE 2:**
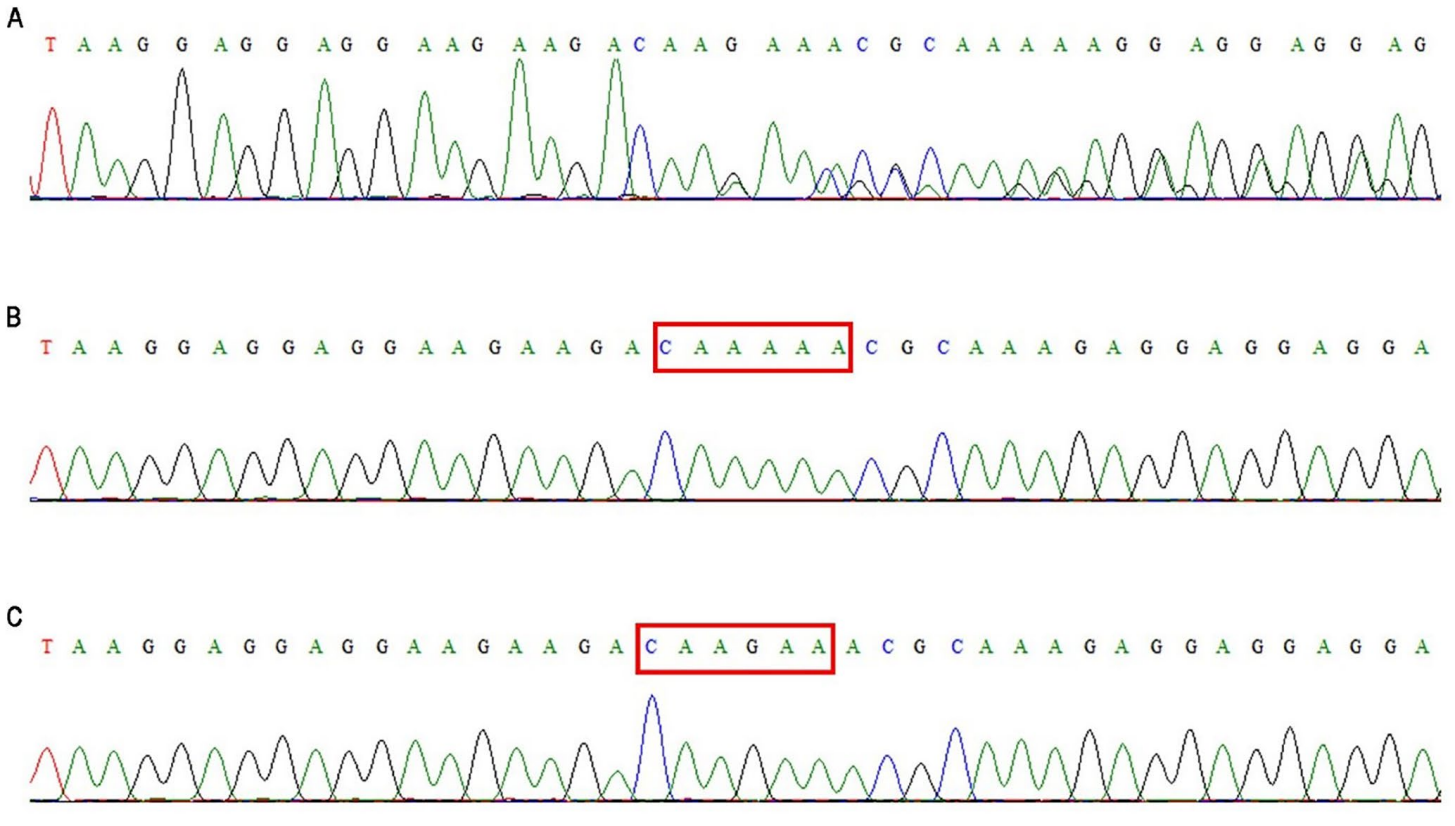
Verification of the *CALR* variant by PCR amplification and Sanger sequencing. (A) Sanger sequencing reveals a frameshift mutation in the *CALR* gene. (B) Cloning and sequencing of PCR products confirm the presence of a mutated clone with the c1122delG deletion. (C) Cloning and sequencing also identify a wild‐type clone, verifying the heterozygous nature of the mutation.

This case underscores critical challenges in diagnosing and managing MPNs, primarily due to the limitations of current diagnostic tools. Most standard kits are mainly focused on identifying the two common *CALR* mutations, Type 1 and Type 2, leaving rarer mutations often undetected. This gap can lead to misdiagnoses or delays, adversely affecting risk assessment and treatment planning.

Although sequencing methods such as Sanger sequencing and NGS remain the gold standard for the comprehensive detection of *CALR* mutations due to their high sensitivity and specificity, these approaches are labor‐intensive, costly, and not routinely implemented in all clinical settings. Therefore, there is a pressing need to update and expand the coverage of standard diagnostic assays to include a broader spectrum of *CALR* mutations. Enhanced diagnostic tools could improve early and accurate detection, reduce the risk of misdiagnosis, and ultimately optimize clinical management and outcomes for patients with MPN*s*.

In conclusion, to the best of our knowledge, this report presents a rare, documented case of a patient with the Type 26 *CALR* mutation. This finding enhances our understanding of the genetic landscape of MPNs and underscores the importance of comprehensive molecular diagnostics. Further research is needed to better understand the clinical and pathogenic implications of the various *CALR* mutations.

## Funding

The authors have nothing to report.

## Conflicts of Interest

The authors declare no conflicts of interest.

## Data Availability

The data that support the findings of this study are openly available in NCBI SRA database at https://www.ncbi.nlm.nih.gov/nuccore/PV153582, https://www.ncbi.nlm.nih.gov/nuccore/PV153583, reference number PV153582, reference number PV153583.
